# Great expectations: Specific lexical anticipation influences the processing of spoken language

**DOI:** 10.1186/1471-2202-8-89

**Published:** 2007-10-26

**Authors:** Marte Otten, Mante S Nieuwland, Jos JA Van Berkum

**Affiliations:** 1 Department of Psychology, University of Amsterdam, The Netherlands; 2 Department of Psychology, Tufts University, Medford, MA, USA; 3 MGH/MIT/HMS Athinoula A. Martinos Center for Biomedical Imaging, Charlestown, MA, USA; 4 Max Planck Institute for Psycholinguistics, Nijmegen, The Netherlands; 5 F.C. Donders Centre for Cognitive Neuroimaging, Nijmegen, The Netherlands

## Abstract

**Background:**

Recently several studies have shown that people use contextual information to make predictions about the rest of the sentence or story as the text unfolds. Using event related potentials (ERPs) we tested whether these on-line predictions are based on a message-level representation of the discourse or on simple automatic activation by individual words. Subjects heard short stories that were highly constraining for one specific noun, or stories that were not specifically predictive but contained the same prime words as the predictive stories. To test whether listeners make specific predictions critical nouns were preceded by an adjective that was inflected according to, or in contrast with, the gender of the expected noun.

**Results:**

When the message of the preceding discourse was predictive, adjectives with an unexpected gender inflection evoked a negative deflection over right-frontal electrodes between 300 and 600 ms. This effect was not present in the prime control context, indicating that the prediction mismatch does not hinge on word-based priming but is based on the actual message of the discourse.

**Conclusion:**

When listening to a constraining discourse people rapidly make very specific predictions about the remainder of the story, as the story unfolds. These predictions are not simply based on word-based automatic activation, but take into account the actual message of the discourse.

## Background

"In this branch house of ours, Handel, we must have a –"

I saw that his delicacy was avoiding the right word, so I said, "A clerk."

"A clerk. And I hope it is not at all unlikely that he may expand into a partner. "

[Great Expectations, Charles Dickens]

In this short exchange, we can see that Pip, the main character of the novel (who is addressed here as Handel by his good friend Herbert), generates not only great expectations but small ones as well. The expectation at hand does not refer to his hopes and plans for the future. Pip merely anticipates how the sentence that his friend is hesitantly uttering will end. This form of prediction – the temptation to finish a slow speaker's sentence- is one we are probably all familiar with in everyday life. Recent event related potential (ERP) studies have shown that predictive processes in language comprehension are not limited to instantiations where the speaker falters. Predictions about the continuation of a sentence or story are actually made regularly and on the fly [1-3]. One important issue is whether these predictions are initiated by a relatively simple automatic activation process, based on (a combination of) individual words in the discourse, or whether they are based on a more thorough understanding of the message of the discourse. In this spoken language ERP experiment we explored which of these constraints actually trigger specific lexical predictions.

Although strong and influential arguments have been made against anticipation in language processing based on the inherent open-ended character of language [cf. [4]], a multitude of psycholinguistic experiments suggests that people do use context to form expectations about the language utterance that is still to follow. These predictive processes pertain to the grammatical role of words [5,6] but also to inferences about the general syntactic [7,8] and semantic [9-17] content of the utterance (see also [18] for a short review). Recent ERP studies have shown that people furthermore use their rapid syntactic and semantic analysis of the discourse to anticipate *specific *words, in spoken [1,19] as well as written language [2,3,20].

DeLong and colleagues [3] have shown that these specific lexical predictions are stronger as the context is more constraining. This contextual constraint, however, can have its predictive effect at two different levels. Predictions could arise from a relatively simple priming process, by which individual words activate lexical-semantic and world knowledge in semantic memory [3,21]. On the other hand, it is well known that our understanding of spoken or written language does not rely on a compilation of disjoint words: we form a comprehensive structured model of the discourse [22-24], combining contextual information through rapid syntactic and semantic analysis [25,26], which includes not only the local but also the wider context [27-29]. Van Berkum and colleagues [1] have therefore suggested that it is more likely that specific lexical predictions are based on an extensive, message-level representation of the discourse than on automatic activation by (a set of) individual words.

To test whether specific lexical predictions are based on the actual message of the discourse or related to some simpler form of word-based priming, we designed predictive stories as well as so-called prime control stories. The predictive stories had a message-level content that supported the prediction of a specific Dutch noun. In the predictive story in Table 1, for example, the word "cross" would indeed be the most sensible and 'expected' continuation at that point, (confirmed by the fact that in a completion test, the large majority of Dutch readers would use "cross" to continue the story at this point). However, note that words like "religious" and "grandparents" are themselves also (mildly) related to "cross", via simple lexical associations (religious – cross) and possibly also scenario-mediated associations (religious grandparents – cross).

**Table 1:** Example of stimulus materials

**Predictive Context**	**Prime Control Context**
Mijn opa en oma zijn erg religieus. Boven hun bed hangt een [kruis]	Mijn opa en oma zijn niet erg religieus. Boven hun bed hangt een [...]
(1) **groot **en nogal dramatisch *kruis*	(1) **groot **en nogal dramatisch *kruis*
(2) **grote **en nogal dramatische *crucifix * aan de muur, en verscheidene schilderijen van heiligen.	(2) **grote **en nogal dramatische *crucifix * aan de muur, maar dat is een erfstuk.
	
*My grandfather and grandmother are very religious. Above the head of their bed hangs a [cross_*neu*_]*	*My grandfather and grandmother are not very religious. Above the head of their bed hangs a [...]*
*(1) ****big*_*neu*_*** and rather dramatic *cross	*(1) ****big*_*neu*_*** and rather dramatic *cross
*(2) ****big*_*com*_*** and rather dramatic *crucifix *on the wall, together with several paintings of saints*.	*(2) ****big*_*com*_*** and rather dramatic *crucifix *on the wall, but that is a family heirloom*.

Example story in the original Dutch version and an approximate English translation, across all four conditions. Critical adjectives are printed in bold face, and critical nouns are printed in italics (in the Dutch example) or regular letters (English example). The word between brackets indicates the predictable word at that point in the story.

To uncover the potential contribution of such simpler priming mechanisms to discourse-based lexical prediction, the prime control stories contained the same potential prime words as the predictive stories but had a completely different and much less predictive message-level representation. As illustrated in the prime control example in Table 1, neither the previously expected noun ("cross") nor the previously less expected noun ("crucifix") is particularly expected (nor, in fact, is any other word) but the possible prime words (i.e. "grandparents" and "religious") are still present in the preceding context sentence. Thus the message-level constraint of the context is low for the prime control context, but the prime-based constraint is identical for the predictive and the prime control context.

To probe whether readers actually predicted the expected noun *before *it came along, we first presented a gender-inflected adjective, with a gender that was consistent or inconsistent with the discourse-predictable noun. In Dutch, adjectives in indefinite noun phrases have a suffix that depends on the arbitrary, lexically memorized gender [30] of the noun they precede. Adjectives that modify a common-gender noun carry an -e suffix (e.g., "grote crucifix", "big_com _crucifix_com_"), whereas adjectives modifying a neuter-gender noun are not inflected (e.g., "groot kruis", "big_neu _cross_neu_").

If listeners strongly anticipate a specific noun, an adjective with a mismatching gender suffix will come as an 'unpleasant' surprise compared to the prediction-consistent adjective. As in previous studies that have employed probes (gender-inflected adjectives in Dutch [1], gender-marked articles in Spanish [2,19,20] and the a/an distinction in English [3]) to test for prediction, we expected that adjectives with an inconsistent adjective inflection would elicit a different ERP effect compared to consistent adjectives. The exact electrophysiological consequences of a prediction mismatch, however, have not been clearly established. Phonological or gender-related information that mismatches a prediction can elicit an increase in N400 amplitude [3,20]. However, prediction mismatches can also elicit negative ERP effects with a timing and scalp distribution that clearly differs from a standard N400 [19,31], as well as positive ERP effects [1,2]. Because the sources of this variability are as yet not understood, the exact nature of the ERP effect to a prediction mismatch was difficult to predict. However, since the majority of the experiments have yielded negative ERP effects as a response to information that (implicitly) contradicts a prediction, it seemed most likely that unexpectedly inflected adjectives would also elicit a more negative ERP. If the differential ERP effect elicited by a prediction-inconsistent adjective inflection in the predictive condition is solely based on word-word priming, we should observe the same effect in the prime control condition. On the other hand, if the lexical prediction effect in predictive stories critically hinged on the entire message conveyed by the discourse up to that point, no such effect should be observed in prime control stories.

## Results

**Figure 1:**
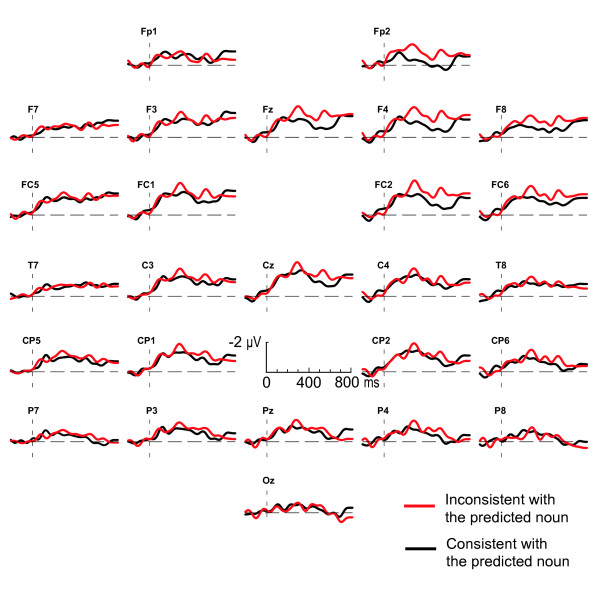
**Adjectives in a predictive context**. Grand average ERPs elicited by the critical adjectives in a predictive context. Black lines represent the response to adjectives bearing an inflection that is consistent with the gender of the predicted noun; red lines represent responses to gender inconsistent adjectives. The ERPs are timelocked to the onset of the adjective, and are filtered (8 Hz high cut-off, 48 dB/oct) for presentation purposes only. Note that in this and all following figures, negative polarity is plotted upward.

**Figure 2:**
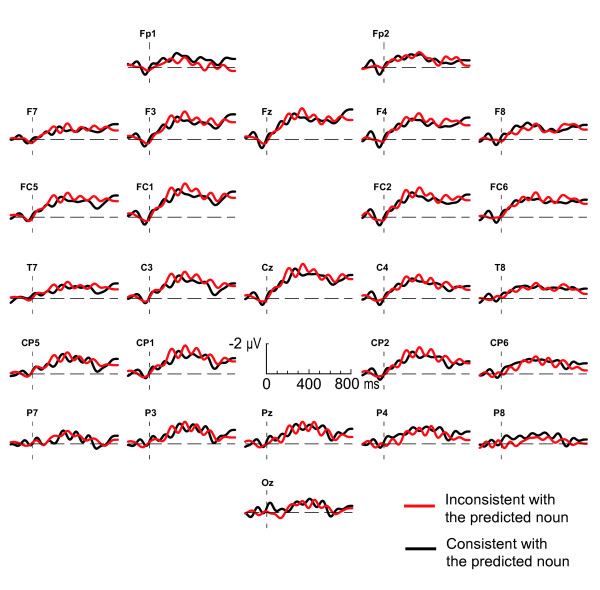
**Adjectives in a prime control context**. Grand average ERPs elicited by the critical adjectives in a prime control context. Black lines represent the response to adjectives bearing an inflection that is consistent with the gender of the predicted noun; red lines represent responses to gender inconsistent adjectives. The ERPs are timelocked to the onset of the adjective, and are filtered (8 Hz high cut-off, 48 dB/oct) for presentation purposes only.

Figure 1 shows the ERPs by prediction-consistent and prediction-inconsistent adjectives in a predictive context, timelocked to the onset of the inflected adjective. Adjectives carrying an inflection inconsistent with the gender of the expected noun evoke a negativity on the right frontal electrodes compared to consistent adjectives, starting at about 300 ms and lasting until 600 ms after the onset of the adjective. Crucially, when the inconsistent adjectives are presented in a prime control context, as depicted in Figure 2, they do not elicit this right-frontal negativity, nor any other differential effect.

The message-level impact of the discourse on the electrophysiological consequences of the implicit mismatch with the gender of the predicted word is reflected by a significant interaction between consistency, context type and electrode quadrant (F(1, 28) = 5.7; p = .02) between 300 and 600 ms. Post-hoc tests for this time-interval show that the interaction between context and consistency is only present in the right frontal quadrant, with the unexpectedly inflected adjectives differing from the expected adjectives in the predictive context (F(1, 28) = 4.5; p = .04) and not in the prime control context (F(1, 28) = 0.1; p = .76)

**Figure 3:**
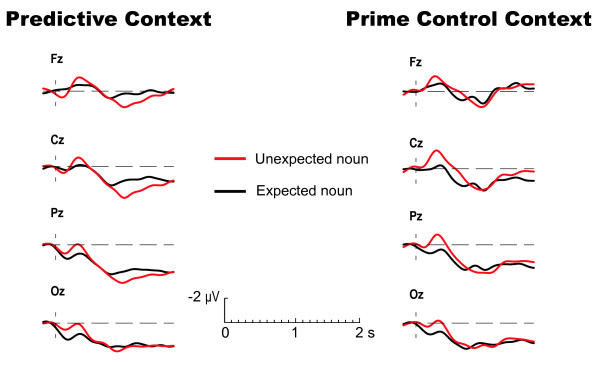
**Nouns in a predictive and prime control context**. Grand average ERPs elicited by the critical nouns in a predictive context and prime control context over the midline electrodes. Black lines represent the ERP to the predicted nouns; red lines represent the ERP to unexpected but still congruent nouns. The ERPs are timelocked to the onset of the noun, and are filtered (4 Hz high cut-off, 48 dB/oct) for presentation purposes only.

For completeness, Figure 3 shows the ERPs elicited by the nouns that follow the critical adjectives in both types of context. Unexpected nouns that follow a predictive context evoke a larger N400 between 200 and 600 ms, as well as a positivity that emerges at around 900 ms, and remains until 1600 ms after word onset. When the same nouns follow a prime control context the N400 effect is still present, but this effect is not followed by a later positive deflection.

The amplitude of the N400 differs significantly for expected and unexpected nouns between 200 and 600 ms (F(1,28) = 7.3; p = .01). The effect does not reliably differ between predictive and prime control context (F(1,28) = 1.6; p = .22). The later widespread positive component elicited by unexpected nouns is reflected in a significant interaction between expectedness and context-type (F(1,28) = 4.7; p = .04) between 1000 and 1500 ms. Post-hoc tests show that this positive shift is only present in the predictive stories (F(1,28) = 10.3; p = .003), and not in the prime control stories (F(1,28) = 0.6; p = .82).

## Discussion

After listening to a constraining discourse whose message suggests a plausible upcoming noun, an adjective with an inflection that is not in line with the gender of the predictable noun elicits a differential ERP effect compared to the adjectives that are consistent with the gender of this noun. Importantly, at this point in the story both gender-inflections are semantically and syntactically correct, since no noun has been shown yet. This ERP effect can therefore only be attributed to a mismatch of the observed gender with the gender of the predictable noun, indicating that listeners have already activated (the gender of) the word they think will follow. This finding thus confirms earlier claims that people use the cues provided by the sentential context or wider discourse to anticipate upcoming words [1-3,7,19,20].

Crucially, the effect of prediction mismatch was absent in the prime control context, which contained the same prime words but did not support a lexical prediction at the message level. This shows that specific lexical predictions of the type observed here are not based on a simple word-based automatic priming process, but critically rely on the precise message-level content. In other words, it is the exact message that counts here, and not the compilation of individual words. Note that we did not specifically include strong primes into the discourse. We therefore can not exclude that in the presence of such primes, prime-induced predictions can also arise independently of the message of the surrounding discourse. The present results, however, clearly show that stories of the type used here induce predictions that are based on the actual message of the preceding context.

The observed electrophysiological consequence of a prediction mismatch resembles other effects of prediction mismatch that have been reported in previous studies in polarity and timing and, to a lesser extent, scalp distribution [3,19,20]. Although our effect resembles the standard N400 effect in timing and polarity, the scalp distribution of the prediction mismatch effect does not resemble the standard distribution of the centro-parietal N400 effect. Therefore, we are reluctant to interpret the present effect as a canonical N400 effect. At the same time, though, the timing of the ERP effect and the critical involvement of high-level meaning are consistent with the idea that at least some of the neural generators that underlie the canonical N400 effect might also be activated when people hear something that (indirectly) mismatches their prediction.

In addition to these central results, Figure 3 shows that unexpected nouns evoked a larger N400 followed by a relatively long-lasting positive shift in the predictive context, compared to expected nouns. In the prime control condition the N400 effect was also present, whereas the late positive shift disappeared.

Within the domain of language processing, late positive components are often related to syntax-based reanalysis [32,33]. However, since the unexpected nouns in the present experiment are not incongruous at any level, they are not very likely to induce re-analysis of earlier syntactic assignments. An alternative possibility might be that the observed positivity reflects the processing of improbable events [34]. What this leaves to be explained, however, is why a similar late positivity was not observed in other studies with semantically unexpected words [e.g. [35]].

In contrast to the late positivity, the N400 is present in both predictive and prime control context. This pattern of results seems to suggest that the N400 does not reflect message-level expectancy or integration, but rather integrative or predictive processes related to word-based priming. However, results from a recent experiment where participants were presented the same stories without the critical preceding adjectives [36] suggest that the discourse-based N400 effect cannot be solely attributed to processes reflecting automatic activation.

A possible explanation for the currently equivalent N400 effects in predictive and prime control stories might lie in the design of the stimuli. In the present experiment the unexpected nouns differ not only from the expected nouns in their level of expectancy and contextual fit, but also in their length and frequency. Hence, the larger N400 for unexpected nouns in both predictive and prime control context could to some extent be attributed to other factors than message-level expectancy.

Furthermore, the cloze values used in this experiment reflect the expectancies that readers or listeners have right at the indefinite article. The adjectives, however, contain additional cues to the nature of the noun that might follow, which will critically alter expectations. As a result, the interpretation of the ERP effects evoked by the nouns is necessarily tentative.

## Conclusion

We have shown that listeners use the information from the context to make predictions about what is to come next, confirming previous research on specific lexical prediction. Furthermore, in a natural discourse these predictions are not based on simple automatic activation processes, but on the exact message of the discourse. People are thus not only capable of rapidly extracting the full meaning of a discourse, but they can also use this knowledge to anticipate what might come next in the story, down to the level of specific upcoming words.

## Methods

### Participants

32 right-handed native speakers of Dutch participated in the experiment as part of a course requirement. Three participants were excluded from analysis because more than 50% of the critical trials had to be deleted due to artefacts (see below). Of the remaining 29 participants 17 were male. Mean age over participants was 23 years, ranging from 18 to 33.

### Materials

The critical stimuli were 160 naturally spoken two-sentence mini-stories, consisting of a context sentence followed by the target sentence. For each item we created a predictive context sentence, that was constraining at a message level, as well as a prime control context sentence, that contained the same prime words but was *not *predictive at the message level. We employed several different strategies in creating the prime control sentences, which are based on the original, predictive, sentence. A selection of stimuli in Appendix 1 illustrates these different strategies: negation (see also the example stimulus in Table 1), adding words, deleting/replacing (non-prime) words, or changing the order of the words.

In a pencil-and-paper "cloze test", 66 participants were shown the stories up to and including the indefinite determiner, and were asked to finish these stories. At least 50 % of the participants used the same noun when the context was predictive, resulting in an average cloze value of 0.74 for the predicted noun (sd = 0.14, ranging from .53 to 1.00) across all predictive stories. For the non-predictive prime control version the response percentage for predicted noun, or any other alternative, was below 30 %, which on average resulted in a cloze value of 0.18 (sd = 0.15, ranging from 0.00 to 0.30). The cloze value for the unexpected target word was 0.03 in both the predictive (sd = .06) and the prime control stories (sd = .07).

In both the predictive and the prime control condition the target sentence could contain the predicted word or an unexpected but still completely coherent alternative. The critical stimuli in this experiment, however were not the (un)expected nouns, but the gender-inflected adjectives that preceded each critical noun. In Dutch indefinite noun phrases, adjectives that modify a common-gender noun take an -e inflection, whereas adjectives that modify a neuter-gender noun take no overt inflection.

Adjectives could be consistent or inconsistent relative to the gender of the predicted noun, but at the time that listeners heard these adjectives both variants of the adjective did not pose an overt violation. Furthermore, to avoid grammatical violations later in the sentence, prediction-inconsistent adjectives were always followed by a coherent but much less expected alternative noun, with a gender that matched the inflection. Across the 160 items, 98 expected nouns had common gender, and 62 had neuter gender. At least 3 words separated the first critical adjective from the (un)expected noun (a second adjective and at least 2 words separating first and second adjective).

Expected and unexpected nouns were not exactly matched on length or frequency. The mean length of the expected and unexpected noun was respectively 6.1 (sd = 2.3) characters and 7.4 characters (sd = 2.3), and the mean frequency for expected and unexpected nouns was respectively 32.2 (sd = 53.7) and 26.7 (sd = 96.0) per 1 million, as stated in the Celex database. A list with all critical items (in Dutch) can be obtained from the first author.

Each story was recorded in four different versions (predictive context – expected inflection/noun, predictive context – unexpected inflection/noun, prime control context – expected noun, prime control context – unexpected noun), by the same female speaker, at normal rate and intonation. The average duration of the critical words was 513 ms for the adjective (range 243 – 924 ms) and 524 ms for the noun (range 170 – 990 ms). The onset of the noun was separated from the onset of the first adjective by 1580 ms on average (range 791–2725 ms). The end of the sentence on average came 2751 ms after the onset of the critical noun (range 1483 – 6070 ms).

Four different trial lists were used. The first list was created by pseudorandomly mixing the 160 critical items (40 for each of the four conditions shown in Table 1) with 90 filler items, so that each participant heard all the stimuli in only one condition. Three more lists were created by rotating the conditions in the original first list.

### Procedure and EEG recording

The total of 250 items were divided in 10 blocks, separated by a pause. Each trial was separated from the next by a 5 sec silence and was preceded by a short warning tone. Total time-on-task was approximately eighty minutes. Participants were seated in front of two loudspeakers, and were informed that they would be listening to short stories. They were instructed to listen for comprehension and minimize movement. No additional task demands were imposed.

The electroencephalogram (EEG) was recorded from 30 electrode sites (FP1, FP2, F9, F7, F3, Fz, F4, F8, F10, FT9, FC5, FC2, FC6, FC1, FT10, T7, C3, Cz, C4, T8, CP5, CP1, Cp2, Cp6, P7, P3, Pz, P4, P8 and Oz), mounted in an elastic cap, each referenced to the left mastoid. Blinks and vertical eye-movements were registered by placing an electrode under the left eye, also referenced to the left mastoid. The EEG was amplified with BrainAmps amplifiers (BrainProducts, München), band-pass filtered at 0.03 Hz-100 Hz and sampled with a frequency of 500 Hz. The EEG signals were re-referenced off-line to the average of right and left mastoids. Blinks and eye movements were removed from the data using a procedure based on Independent Component Analysis (ICA) as described by Jung et al. [37,38].

We timelocked the ERPs to the onset of the critical adjective and noun. After baseline correcting (by subtraction) the waveforms of the individual trials relative to the relevant 200-ms pre-stimulus baseline intervals, we computed average waveforms for each subject and condition relative to the estimated acoustic onset of the first adjective and the noun that followed. Because the earliest (un)expected nouns, signifying an overt (mis)match with the predicted noun, began at about 800 ms after the onset of the critical adjective, we analysed the ERPs evoked by the adjectives in a time-interval from 0 to 800 ms. To avoid spurious effects due to the sentence offset, the window of analysis for the nouns ranged from 0 to 1500 ms after noun onset.

Segments in which the signal exceeded ± 75 μV, or which featured a linear drift of more than ± 50 μV, beginning before the onset of the critical word, were eliminated. For three subjects the data loss exceeded 50% (respectively 67%, 79% and 91%, averaged over all conditions and critical words), and therefore these subjects were excluded from further analysis. For the remaining 29 subjects 23% of the trials was deleted (ranging between subjects from 2% to 48%). The proportion of deleted trials did not differ across conditions.

### Analyses

To assess not only the effects of consistency and context type, but also the possible interaction with electrode position the ERPs elicited by adjectives and nouns were evaluated in an ANOVA crossing Consistency (prediction consistent/prediction-inconsistent), Context (predictive/prime control), Hemisphere (left/right) and Anteriority (anterior/posterior). This analysis thus involved four quadrants: (1) left-anterior, comprising FP1, F3, F7, F9, FC1, FC5 and FT9; (2) right-anterior, comprising FP2, F4, F8, F10, FC2, FC6 and FT10; (3) left-posterior, comprising C3, T7, Cp1, Cp5, P3 and P7; (4) right-posterior, comprising C4, T8, CP2, CP6, P4 and P8. Effects on the midline electrodes (Fz, Cz, Pz and Oz) were assessed in a separate ANOVA crossing the factors Context, Consistency and Electrode position. F tests with more than one degree of freedom in the numerator were adjusted by means of the Greenhouse-Geisser or Huynh-Feldt correction where appropriate. Uncorrected degrees of freedom and corrected P-values are reported.

## Authors' contributions

MO conceived of the study and participated in the design, carried out data collection, EEG analysis and statistical analysis, interpreted the data and drafted the manuscript. MSN participated in the design of the study, carried out data collection and EEG analysis, and helped to draft the manuscript. JJAvB participated in the design and coordination of the study, and helped to draft the manuscript. All authors read and approved the final manuscript.

## Appendix

Four additional example-stories from the stimulus materials, in the original Dutch version and an approximate English translation. These stories are representative of the entire stimulus-set and exemplify the different ways in which each predictive context was changed into a less predictive prime control story.

### Example 1

#### Predictive Context

Nadat hij uren naar het enorme lege doek had gekeken voelde de schilder inspiratie opkomen. Hij greep naar een **grote **vanwege intensief gebruik sleetse kwast/**groot **vanwege intensief gebruik sleets paletmes en smeet de verf op het doek.

*After watching the big empty canvas for hours the painter felt inspiration coming up. He grabbed a big*_*com*_*, and, due to heavy use, very worn brush/big*_*neut*_*, and, due to heavy use, very worn palette-knife and threw the paint on the canvas*.

#### Prime Control Context

Nadat hij uren naar het enorme lege doek had gekeken had de schilder nog steeds geen inspiratie. Hij greep naar een **grote **vanwege intensief gebruik sleetse kwast/**groot **vanwege intensief gebruik sleets paletmes en smeet deze door zijn atelier.

*After watching the big empty canvas for hours the painter still felt no inspiration. He grabbed a ****big*_*com*_***, and, due to heavy use, very worn brush/****big*_*neut*_***, and, due to heavy use, very worn palette-knife and threw it through his studio*.

### Example 2

#### Predictive Context

Anne had eindelijk een rustig plekje gevonden waar ze kon studeren. Ze ging zitten en pakte een **dik **en behoorlijk beduimeld boek/**dikke **en behoorlijk beduimelde roman uit haar tas.

*Anne had finally found a quiet place for studying. She sat down and grabbed a ****big*_*neut*_*** and pretty well-thumbed book/****big*_*com*_*** and pretty well-thumbed novel out of her bag*.

#### Prime Control Context

Na het studeren had Anne een rustig plekje in het park gevonden. Ze ging zitten en pakte een **dik **en behoorlijk beduimeld boek/**dikke **en behoorlijk beduimelde roman uit haar tas.

*After studying Anne had found a quiet place in the park. She sat down and grabbed a ****big*_*neut*_*** and pretty well-thumbed book/****big***_*com*_* and pretty well-thumbed novel out of her bag*.

### Example 3

#### Predictive Context

De misdadiger is opgepakt en veroordeeld en zit voor drie jaar in een gevangenis. Hij zit bijna altijd in een **verouderde **en daarom behoorlijk onprettige cel/**verouderd **en daarom behoorlijk onprettig gevang maar komt binnenkort vrij.

*The criminal has been arrested and sentenced and is now in prison for three years. He spends all his time in an ****old*_*com*_*** and therefore rather unpleasant cell/****old*_*neut*_*** and therefore rather unpleasant jail but he will be out soon*.

#### Prime Control Context

De misdadiger heeft zijn leven gebeterd nadat hij was opgepakt en veroordeeld tot drie jaar gevangenis. Hij zit bijna altijd in een **verouderde **en daarom behoorlijk onprettige cel/**verouderd **en daarom behoorlijk onprettig gevang maar komt binnenkort vrij.

*The criminal has mended his ways after he was arrested and sentenced to prison for three years. He spends all his time in an ****old*_*com*_*** and therefore rather unpleasant cell/****old*_*neut*_*** and therefore rather unpleasant jail but he will be out soon*.

### Example 4

#### Predictive Context

Het kleine kind had het warm vanwege de hittegolf en liep te zeuren. Ze wilde een **koud **en liefst ook lekker ijsje/**koude **en liefst ook lekkere ijslolly om af te koelen.

*Because of the hot weather the little girl was warm and whiney. She wanted a ****cold*_*neut*_*** and preferably also tasty ice cream/****cold*_*com*_*** and preferably also tasty popsicle to cool down a bit*.

#### Prime Control Context

De moeder had het warm vanwege the hittegolf en vond dat haar kind liep te zeuren. Ze wilde een **koud **en liefst ook lekker ijsje/**koude **en liefst ook lekkere ijslolly om af te koelen.

*Because of the hot weather the mother was warm and thought her little girl was whiney. She wanted a ****cold*_*neut*_*** and preferably also tasty ice cream/****cold*_*com*_*** and preferably also tasty popsicle to cool down a bit*.
